# Identification and Characterization of Immunodominant Proteins from Tick Tissue Extracts Inducing a Protective Immune Response against *Ixodes ricinus* in Cattle

**DOI:** 10.3390/vaccines9060636

**Published:** 2021-06-10

**Authors:** Sarah Knorr, Sophia Reissert-Oppermann, Julen Tomás-Cortázar, Diego Barriales, Mikel Azkargorta, Ibon Iloro, Félix Elortza, Sophia Pinecki-Socias, Juan Anguita, Joppe W. Hovius, Ard M. Nijhof

**Affiliations:** 1 Institute for Parasitology and Tropical Veterinary Medicine, Freie Universität Berlin, 14163 Berlin, Germany; knorr.sarah.j@gmail.com (S.K.); sophia.reissert@googlemail.com (S.R.-O.); Sophia.Pinecki@fu-berlin.de (S.P.-S.); 2 School of Biomolecular and Biomedical Science, University College Dublin, Belfield, D04 V1W8 Dublin 4, Ireland; julen.tomascortazar@ucd.ie; 3 CIC bioGUNE-BRTA (Basque Research and Technology Alliance), 48162 Derio, Spain; dbarriales@cicbiogune.es (D.B.); mazkargorta@cicbiogune.es (M.A.); iiloro@cicbiogune.es (I.I.); felortza@cicbiogune.es (F.E.); janguita@cicbiogune.es (J.A.); 4 Ikerbasque, Basque Foundation for Science, 48009 Bilbao, Spain; 5 Center for Experimental and Molecular Medicine, Academic Medical Center, University of Amsterdam, 1105 AZ Amsterdam, The Netherlands; lyme@amc.uva.nl

**Keywords:** *Ixodes ricinus*, immunoprecipitation, anti-tick vaccines, midgut, salivary glands

## Abstract

*Ixodes ricinus* is the main vector of tick-borne diseases in Europe. An immunization trial of calves with soluble extracts of *I. ricinus* salivary glands (SGE) or midgut (ME) previously showed a strong response against subsequent tick challenge, resulting in diminished tick feeding success. Immune sera from these trials were used for the co-immunoprecipitation of tick tissue extracts, followed by LC-MS/MS analyses. This resulted in the identification of 46 immunodominant proteins that were differentially recognized by the serum of immunized calves. Some of these proteins had previously also drawn attention as potential anti-tick vaccine candidates using other approaches. Selected proteins were studied in more detail by measuring their relative expression in tick tissues and RNA interference (RNAi) studies. The strongest RNAi phenotypes were observed for MG6 (A0A147BXB7), a protein containing eight fibronectin type III domains predominantly expressed in tick midgut and ovaries of feeding females, and SG2 (A0A0K8RKT7), a glutathione-S-transferase that was found to be upregulated in all investigated tissues upon feeding. The results demonstrated that co-immunoprecipitation of tick proteins with host immune sera followed by protein identification using LC-MS/MS is a valid approach to identify antigen–antibody interactions, and could be integrated into anti-tick vaccine discovery pipelines.

## 1. Introduction

*Ixodes ricinus* is a three-host ixodid tick species that is widely distributed in Western Europe. It is the predominant vector of several pathogens of medical and veterinary relevance, including tick-borne encephalitis virus (TBEV), *Borrelia burgdorferi* sensu lato (the causal agent of Lyme borreliosis), and *Babesia divergens* [1]. Besides their capacity to transmit a wide variety of pathogens, it was recently demonstrated that the saliva of *I. ricinus* contained galactose-α-1,3-galactose (α-Gal)-carrying proteins, which are associated with the induction of an anti-α-Gal immune response in humans and may result in red meat allergy [2,3]. The prevention and control of diseases associated with *I. ricinus* relies on a combination of methods including the avoidance of tick habitats, the prompt removal of ticks, the use of repellents and acaricides, and landscaping measures [4].

Vaccines targeting ticks and the blocking of pathogen transmission are attractive alternative control options, and their development is drawing increasing interest [4,5,6,7]. Anti-tick vaccines targeting the common cattle tick *Rhipicephalus microplus* have been successfully developed and commercialized in the last century [8,9]. These vaccines are based on a single recombinant antigen, Bm86, a protein located in the microvilli of the tick midgut. This protein was identified following a laborious and long process involving multiple immunization trials, in which the effect of immunization with increasingly simpler fractions of tick midgut extracts on tick infestation was evaluated [10]. The artificial feeding of blood and plasma of animals immunized with tick midgut extracts showed that antibodies, in the presence of complement, were able to damage tick gut cells, thereby reproducing some of the detrimental effects observed in *R. microplus* ticks fed directly on immunized cows [11]. Antisera raised against recombinant Bm86 also partially inhibited larval engorgement of one-host *R. australis* (formerly *R. microplus*) ticks [12]. Our group recently immunized calves with different organ tissue homogenates of *I. ricinus* and found that the immunization with the soluble extracts of all tick organs, salivary glands, or midgut alone also conferred significant protection against subsequent challenge with *I. ricinus* nymphs and adults [13]. The identification of antibody–antigen complexes responsible for disrupting tick feeding could therefore be of relevance in the identification of tick-protective antigens.

In this study, we co-immunoprecipitated tick tissue extracts with pre- and post-immune sera from the *I. ricinus* immunization trial followed by label-free liquid chromatography–mass spectrometry (LC-MS/MS) to identify proteins that were differentially recognized by the serum of calves immunized with salivary gland extracts (SGE) or midgut extracts (ME). The results were partially validated by Western blotting, and the gene expression profile of selected antigens in tick organs was measured by quantitative reverse transcription PCR (RT-PCR). Finally, we analyzed the loss-of function phenotype for 10 of the identified proteins by RNA interference (RNAi).

## 2. Materials and Methods

### 2.1. Ticks and Animals

All *I. ricinus* ticks used originated from a laboratory colony maintained at the tick breeding unit of the Institute for Parasitology and Tropical Veterinary Medicine of the Freie Universität Berlin.

### 2.2. Protein Extracts and Antisera

Salivary glands and midguts were dissected from washed ticks and kept in sterile PBS on ice. The tissues were subsequently homogenized using an ultrasonic homogenizer (Hielscher, UP100H) and centrifuged at 15,000× *g* for 30 min at 4 °C. The supernatant was filtered through 0.4 µm and 0.2 µm non-pyrogenic filters (Sarstedt, Nümbrecht, Germany) and stored at −20 °C. The bovine antisera used originated from calves immunized with tick protein extracts, as recently described [13]. Antisera were collected at day 0 (pre-immune sera) and day 68 (post-immune sera).

### 2.3. Direct Antigen Co-Immunoprecipitation

Control and immune calf sera IgG were purified using the Melon Gel IgG Spin Purification Kit (Thermo Scientific, Rockford, IL, USA), following the manufacturer’s protocol. Protein content was then measured in midgut and salivary gland lysates using the Pierce BCA Protein Assay Kit (Thermo Scientific, Rockford, IL, USA). Direct immunoprecipitation was performed using the Pierce Direct IP Kit (Thermo Scientific, Rockford, IL, USA) following the manufacturer’s instructions. Enrichment was carried out by incubation of 100 μL of the AminoLink Plus Coupling Resin slurry with 20 μL purified serum that was immobilized onto the aldehyde-activated agarose resin at room temperature for 2 h. One milligram of the tissue lysates was added to each antibody-coupled resin in a spin column. The column was incubated with gentle shaking at 4 °C overnight to form antibody–antigen complexes. After several washes, the antigens were eluted in 100 μL.

### 2.4. Tryptic Digestion

SDS-PAGE bands of the immunoprecipitated antigens were cut and washed in milli-Q water. Reduction and alkylation were performed using ditiothreitol (10 mM DTT in 50 mM ammonium bicarbonate) at 56 °C for 20 min, followed by iodoacetamide (50 mM iodoacetamide in 50 mM ammonium bicarbonate) at room temperature for another 20 min in the dark. Gel pieces were dried and incubated with trypsin (12.5 μg/mL in 50 mM ammonium bicarbonate) for 20 min on ice. After rehydration, the trypsin supernatant was discarded; gel pieces were hydrated with 50 mM ammonium bicarbonate, and incubated overnight at 37 °C. After digestion, tryptic peptides were recovered and dried in an RVC2 25 speedvac concentrator (Christ, Osterode, Germany). The peptides were resuspended in 10 μL 0.1% formic acid and sonicated for 5 min prior to analysis.

### 2.5. LC-MS/MS Analysis

LC was performed using an NanoAcquity nano-HPLC (Waters, Milford, MA, USA) apparatus equipped with a Waters BEH C18 nano-column (200 mm × 75 um ID, 1.8 µm). A chromatographic ramp of 120 min (5 to 60% ACN) was used with a flow rate of 300 nL/min. Mobile phase A was water containing 0.1% *v*/*v* formic acid, while mobile phase B was ACN containing 0.1% *v*/*v* formic acid. A lock mass compound, [Glu1]-Fibrinopeptide B (100 fmol/µL), was delivered by an auxiliary pump of the LC system at 500 nL/min to the reference sprayer of the NanoLockSpray (Waters) source of the mass spectrometer. For each run, 0.5 µg of each sample was loaded.

For mass spectrometry, we used a Synapt G2Si ESI Q-Mobility-TOF spectrometer (Waters) equipped with an ion mobility chamber (T-Wave-IMS) for high-definition data acquisition analyses. All analyses were performed in positive-mode ESI. Data were post-acquisition lock mass-corrected using the double-charged monoisotopic ion of [Glu1]-Fibrinopeptide B. Accurate mass LC-MS/MS data analysis was performed in HDDA mode, which is an enhanced form of data-dependent acquisition that enhances signal intensities using the ion mobility separation step.

Database searching was performed using MASCOT 2.2.07 (Matrixscience, London, UK) against a custom UNIPROT–Swissprot/Trembl database filled only with entries corresponding to *Ixodes* and *B. burgdorferi*. For protein identification, the following parameters were adopted: carbamidomethylation of cysteines (C) as a fixed modification and oxidation of methionines (M) as variable modifications, 15 ppm of peptide mass tolerance, 0.2 Da fragment mass tolerance, up to 3 missed cleavage points, and peptide charges of +2 and +3. Only peptides with a false discovery rate <1% were selected.

### 2.6. Cloning and Purification

The MG9 (A0A131YAQ2) and SG4 (A0A0K8RQF1) genes were cloned by overlapping PCR from midgut or salivary gland cDNA and cloned as EcoRI-XhoI or NcoI-SalI fragments, respectively, into the pHIS-parallel 2 expression vector. Sequence-confirmed clones were induced with 1 mM isopropyl-β-D-thiogalactoside (IPTG) for 16 h at 20 °C in *E. coli* BL21 C41(DE3). The bacterial cells were then lysed and centrifuged. The expressed proteins were extracted from the inclusion bodies using the following protocol: the pellet was thoroughly homogenized in 50 mM Tris (pH 8), 300 mM NaCl, 1 mM DTT, and 2% Triton X-100, followed by an incubation at 37 °C for 30 min. The sample was ultracentrifuged at 96,000× *g* for 30 min and the pellet was homogenized again in 50 mM Tris (pH 8), 300 mM NaCl, and 1 mM DTT, and incubated at 37 °C for 30 min. After a second ultracentrifugation, the pellet was homogenized in 50 mM Tris pH 8, 300 mM NaCl, 1 mM DTT, and 7 M urea. The denatured proteins were refolded by dialysis in PBS overnight with an intermediate exchange of buffer to a final concentration of 2 M urea.

### 2.7. Western Blotting

For the validation or recognition of tick antigens by bovine immune sera, 5 μg of each extract or purified protein were boiled at 95 °C for 10 min, subjected to SDS-PAGE, and transferred to a nitrocellulose membrane at 200 V for 1 h. The membranes were blocked with 5% non-fat milk in Tris-buffered saline solution containing 0.01% Tween-20 (TBS-T). The membranes were immunoblotted with diluted pre-immunization control sera (d0) and post-immunization sera (d68) (1:500) at 4 °C overnight.

### 2.8. Bioinformatics Analysis

The BLASTp tool was used to infer the potential function by homology. Signal peptides were predicted by SignalP (http://www.cbs.dtu.dk/services/SignalP/, accessed on 15 August 2019). Transmembrane helices were predicted using the TMHMM server (http://www.cbs.dtu.dk/services/TMHMM/, accessed on 15 August 2019).

### 2.9. RNA Isolation

Tissues were dissected from unfed *I. ricinus* females and females pre-fed for 3–5 days on rabbits. Three biological replicates were made for each tissue type. Prior to dissection, ticks were washed for 30 s in 70% ethanol. Dissections were performed on a glass slide under ice-cold phosphate-buffered saline (PBS, pH 7.2). Internal organs were stored in TRI Reagent (Sigma-Aldrich, Taufkirchen, Germany) on ice and homogenized by passage through 24- and 27-gauge needles. Total RNA was subsequently isolated by chloroform phase separation and isopropanol precipitation, followed by DNase treatment (Thermo Fisher Scientific, Darmstadt, Germany). Sample concentrations and purity were measured using a Synergy HT Spectrophotometer (Bio-Tek Instruments, Bad Friedrichshall, Germany).

### 2.10. Quantitative RT-PCR

cDNA was synthesized from 100 ng of DNA-free RNA from the salivary glands, midguts, Malpighian tubules, ovaries, and fat bodies of unfed and partially fed *I. ricinus* females using the iScript cDNA synthesis kit (Bio-Rad laboratories, Feldkirchen, Germany) according to the manufacturer’s instructions and stored at −20 °C. Quantitative RT-PCRs were performed for 15 targets identified in the LC-MS/MS analysis as being differentially recognized by immune sera and for the genes targeted by RNAi (see below). Two references genes, elongation factor 1-alpha (ELF1A) [14] and ATP synthase subunit g (ATP5L), were used for normalization purposes. ATP5L was used as a reference gene as it was recently found to be an abundant and stably expressed gene in *Borrelia afzelii*-infected as well as uninfected *I. ricinus* ticks in a quantitative transcriptomics study [15]. A list of primers used for quantitative RT-PCRs is presented in Table 1. All PCRs were conducted in a Bio-Rad CFX qPCR cycler. RT-PCR amplification mixtures (25 µL) contained 12.5 µL of Advanced Universal SYBR Green Supermix (Bio-Rad Laboratories), 2.5 µL cDNA template, 400 nM of both forward and reverse primer, and 8 µL water. The cycling conditions were 30 s at 95 °C followed by 40 cycles of 95 °C for 10 s and 60 °C for 30 s. A melt curve analysis was performed from 65 to 95 °C with a 0.5 °C increment with 2 s/step. All assays included a no-template control for each gene. Gene expression was analyzed using CFX Maestro software (Bio-Rad).

### 2.11. RNA Interference

For the RNAi experiment, cDNA was synthesized from RNA isolated from tick tissues using the Superscript III first-strand cDNA synthesis kit (Thermo Fisher Scientific, Darmstadt, Germany) according to the manufacturer’s instructions. Oligonucleotide primers (Sigma-Aldrich, Taufkirchen, Germany) containing a T7 promotor sequences at the 5′-end were used to amplify partial fragments of the genes coding for 10 target genes and green fluorescent protein (GFP) (Table 2). PCR products were purified using the DNA Clean and Concentrator kit (Zymo Research, Freiburg im Breisgau, Germany) following the manufacturer’s recommendations and used as templates to produce dsRNA using the T7 Ribomax Express RNAi system (Promega, Walldorf, Germany) according to the manufacturer’s instructions.

In the RNAi experiment, the effect of silencing the expression of 10 genes identified by the LC-MS/MS analysis was evaluated. Ticks were divided into 10 groups of 40 female ticks each that were subsequently injected with 0.5 µL of dsRNA (1 × 10^12^ molecules/µL dissolved in 10 mM Trish-HCl, pH 7 and 1 mM EDTA) coding for one of the selected targets. Female *I. ricinus* ticks were injected in the lower right quadrant using a 10 µL syringe with a 33-gauge needle (Hamilton) mounted on a micromanipulator. As a negative control, 80 ticks were equally divided over two groups and injected with dsRNA coding for GFP. Following injection with dsRNA, ticks were incubated at RT and 90% relative humidity for 24 h. The ticks were thereafter fed on six rabbits, with one group per ear. The two negative control groups injected with GFP dsRNA fed on different rabbits. For the confirmation of gene silencing by quantitative RT-PCR, the RNA from the salivary glands or midguts of six female ticks fed for five days from each group were collected and analyzed in three biological triplicates of two ticks each. Engorged females were weighed individually, and oviposition data were not recorded.

### 2.12. Statistical Analysis

Statistical analysis of data from the weights of ticks after feeding was performed using GraphPad Prism version 5.03 for Windows. The proportion of ticks that successfully engorged were analyzed by chi-squared test. Tick weights were compared between the experimental and combined GFP-injected groups using a *t*-test with Welch’s correction. Quantitative RT-PCR data was analyzed using the Bio-Rad CFX Maestro software. *p*-values of 0.05 or less were considered statistically significant.

## 3. Results

### 3.1. Identification of Differentially Recognized Proteins from ME and SGE by Immune Sera of Calves Immunized with ME and SGE

**Figure 1:**
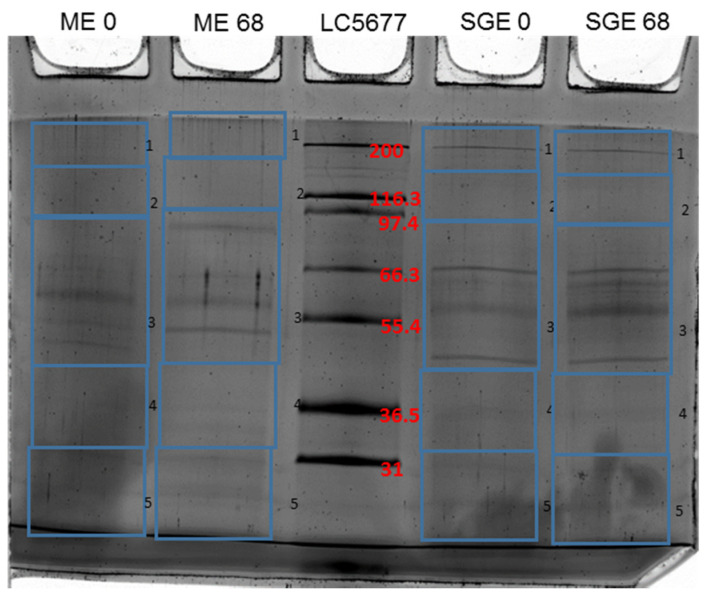
The SDS-PAGE gel of the immunoprecipitation complexes was cut as depicted, followed by in-gel tryptic digestion for the proteomic analysis. Abbreviations—ME: midgut extract; SGE: salivary gland extracts; LC5677: Mark12 protein marker (Invitrogen).

In order to identify immunodominant proteins from SGE and ME, we immunoblotted midgut and salivary gland extracts using pre-immunization control sera (d0) and post immunization sera (d68) from calves. Both sera showed specific recognition of proteins in SGE and ME extracts that were not recognized by control sera (Figure 1).

SGE antisera identified 167 unique peptides which could be linked to 55 proteins in the Uniprot database. Alignment of these proteins with their closest BLASTp hits revealed that four of the Uniprot entries (V5I085/A0A131Y1S2 and V5HWD5/A0A0K8RPW5) actually represented different fragments of two proteins, reducing the total number of uniquely identified proteins to 53. Of these proteins, five were only recognized by control sera and 24 exclusively by immune sera. A total of 24 proteins were identified in SGE-immunoprecipitates with both d0 and d68 sera (Table 3). In the ME, 78 unique peptides were found that could be linked to 46 proteins. Again, four UNIPROT entries (V5H0K4/A0A147BSS4 and A0A131Y7G4/A0A090X8W5) actually represented different fragments of two proteins, reducing the number of unique proteins to 44 proteins. A total of eight proteins were identified in ME-immunoprecipitates with both d0 and d68 sera, while 14 were identified by the control sera, and 22 by the immune sera (Table 4). Finally, 13 proteins were present in both SGE and ME immunoprecipitates, 10 of which in both d0 and d68 sera. A single protein, heat shock protein 60 (A0A131XPM3), was recognized by d68 sera of both SGE and ME immunized calves.

### 3.2. Expression of Recombinant Immunodominant Proteins and Validation by Western Blot

**Figure 2:**
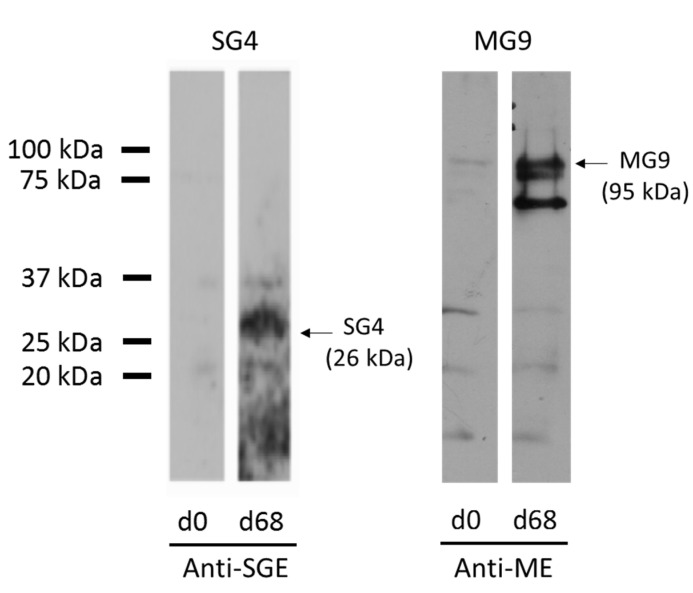
SG4 and MG9 Western blot as validation for the immunoprecipitation employed to identify new vaccine antigens (d0: control sera; d68: immune sera).

Validation of the LC-MS/MS analysis was performed by the recombinant expression of two identified proteins that were differentially recognized by the immune sera: a putative Toll-like receptor (SG4, UNIPROT ID A0A0K8RQF1) from the SGE and an uncharacterized protein (MG9, UNIPROT ID A0A131YAQ2) from the ME. Western blot analysis confirmed the differential recognition of these two proteins (Figure 2).

### 3.3. Expression Profile of Selected Genes in Different Tissues of Ixodes ricinus Females

**Figure 3:**
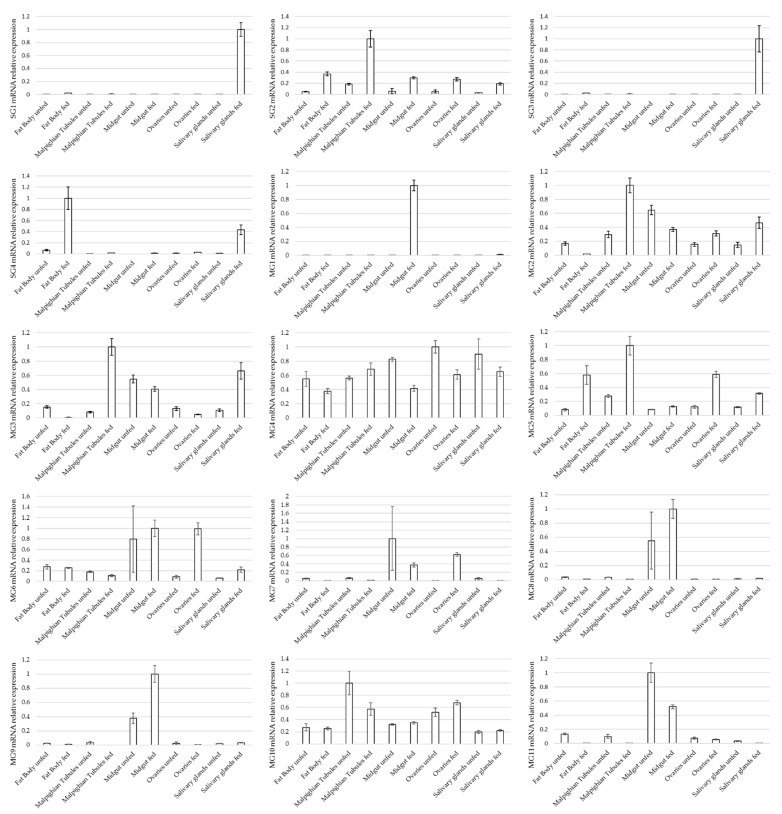
Relative gene expression of selected candidates identified by LC-MS/MS analysis of unfed and partially fed *Ixodes ricinus* females. Bars represent the standard deviation of the normalized gene expression.

Eleven ME proteins and four SGE proteins were subsequently selected for further analysis based on their differential recognition by d68 sera, having putative extracellular exposure or being predicted to be secreted. The expression profile of these proteins was determined by quantitative RT-PCR. The results corroborated findings of the proteomic analysis since proteins identified by LC-MS/MS from SGE and ME immunoprecipitates were expressed in the salivary glands and midguts, respectively. Two salivary gland proteins (SG1 and SG3) that were predicted to be different fragments of the same metalloproteinase had similar expression profiles. A few proteins were expressed in salivary glands or midguts exclusively, such as the metalloproteinase mentioned above and two uncharacterized midgut proteins with unknown homology (MG8 and MG9). However, most of the proteins were expressed in multiple tick tissues (Figure 3).

### 3.4. Effect of Gene Silencing of Selected Candidates on I. ricinus Adult Feeding

**Figure 4:**
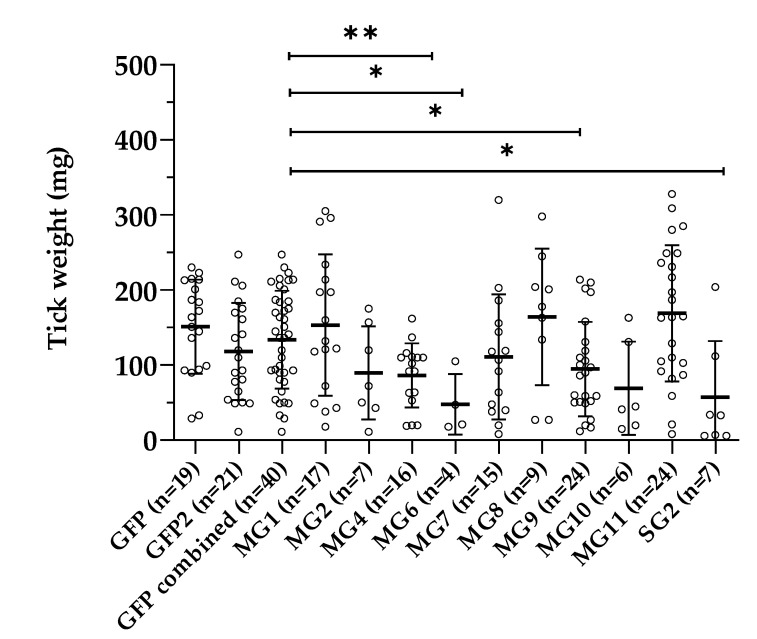
Weight of replete *Ixodes ricinus* females injected with dsRNA coding for GFP (control groups) and 10 gene targets identified by LC-MS/MS. Errors bars represent the standard deviation (SD). The asterixis indicate a significant difference in weight; * (*p* < 0.05), ** (*p* < 0.005).

**Figure 5:**
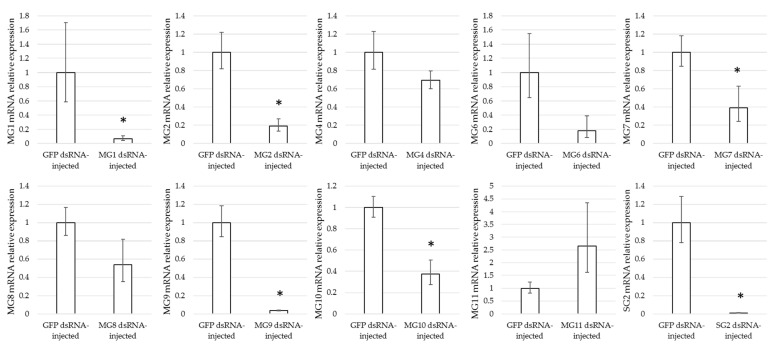
mRNA expression levels of target genes, relative to the expression in GFP-injected ticks. Compared to the control ticks, only the expression of target genes MG1, MG2, MG7, MG9, MG10, and SG2 was significantly knocked down in the respective experimental groups. * indicates a significant difference between the control and experimental group (*p* < 0.05).

Female ticks were injected with dsRNA complementary to GFP (control), SG2, MG1, MG2, MG4, MG6, MG7, MG8, MG9, MG10, or MG11, and were subsequently allowed to feed on rabbits. The proportion of ticks that were engorged was significantly lower for the MG2 (*p* = 0.0003), MG6 (*p* < 0.0001), MG8 (*p* = 0.0026), MG10 (*p* < 0.0001), and SG2 (*p* = 0.0003) groups compared to the GFP control groups. The engorgement weights of both GFP-injected control groups were not significantly different, and the engorgement weights obtained in the experimental groups were compared to the weights of the combined control groups. Significantly lower engorgement weights were found for MG4 (*p* = 0.0025), MG6 (*p*= 0.0139), MG9 (*p* = 0.0213), and SG2 (*p* = 0.0356) (Figure 4). Quantitative RT-PCR showed that the expression of the target genes was silenced in each respective group, with the exception of MG11, where a non-significant increase in MG11 gene expression levels was found in the ticks injected with MG11 dsRNA (Figure 5). Significant gene silencing was found for MG1, MG2, MG7, MG9, MG10, and SG2 (*t*-test, *p* < 0.05). The lower expression levels detected for MG4 and MG6 were not significant.

## 4. Discussion

The main bottleneck in the development of successful anti-tick vaccines is the identification of tick-protective antigens that are effective in limiting tick infestations when applied as recombinant antigens. Several approaches have been followed to identify tick-protective antigens, including studies of the immune response in tick-immune hosts, the evaluation of the increasingly simpler native protein extracts in vaccination, and challenge trials, as well as the identification of antigens that are crucial for the survival or function of ticks, for instance using functional genomic tools such as RNAi [7]. In this study, we used a combination of these approaches, starting with sera of calves immunized with tick extracts that showed a strong immune response upon *I. ricinus* tick challenge, which significantly hampered the feeding of both nymphs and adults [10]. The sera were used for the co-immunoprecipitation of tissue extracts, and differentially recognized proteins were subsequently identified by label-free LC-MS/MS, a step greatly facilitated by the increasing amounts of proteomic and genomic data that have become available for ticks over the last years [16]. Several differentially recognized antigens were subsequently characterized in more detail by determining their tissue expression profiles and loss-of-function phenotype by RNAi.

A considerable number of proteins identified by co-immunoprecipitation together with LC-MS/MS have previously also drawn attention as possible anti-tick vaccine candidates by other approaches. These include homologs of glutathione-S-transferase (SG2) [17,18,19], tropomyosin [20,21], ubiquitin and elongation factor EF1-alpha [22,23], myosin light chain [24], heat shock protein 70 [25], cathepsin L [26], enolase [27], antigen B from *R. microplus* (AAN15115) with 67.4% (663/984 amino acid (AA)) identity to MG8 [28], a glyceraldehyde-3-phosphate dehydrogenase from *Haemaphysalis flava* (AVK70348) with 88.2% (293/332 AA) identity to A0A0K8RG01 [29], and SG1/SG3 (A0A0K8RPW5/V5HWD5), a metalloproteinase with 60.2% (136/226 AA) identity to metis 5 from *I. ricinus* (CAO00629) [30,31].

Glutathione-S-transferases (GSTs) play a role in the excretion of toxic metabolites, and the partially characterized GST SG2 (A0A0K8RKT7) shares 88.3% (197/223) AA identity with DmGSTM1, a mu-class GST from *Dermacentor marginatus* ticks that was recently evaluated as an anti-tick vaccine [17,18]. SG2 also had a similar expression pattern in the tissues of adult females to DmGSTM1 [17]. Gene silencing of GSTs was previously shown to increase the susceptibility of *Rhipicephalus sanguineus* and *Haemaphysalis longicornis* ticks to the ectoparasiticides permethrin and flumethrin, respectively [32,33]. In our study, RNAi-mediated silencing of the expression of SG2 resulted in a smaller proportion of ticks that were engorged, with engorged ticks having significantly lower engorgement weights compared to the control group. Gene silencing of *gst* also significantly reduced the engorgement weights of *R. microplus* females [22], but not of *R. sanguineus* females [32]. These differences may have been caused by differences in the *gst* isoform targeted, experimental procedures, and/or RNAi efficiency.

The second protein that showed a clear loss-of-function phenotype in the RNAi study was MG6 (A0A147BXB7). This ~105 kDa protein is predicted to contain a signal peptide, eight fibronectin type III domains, and a single transmembrane protein. The fibronectin type III domain is one of three types of internal repeats found in fibronectin, a glycoprotein that connects cells to the extracellular matrix, plays a role in cell signaling, and may also act as a target for bacterial adhesion. Fibronectin type III domains also frequently occur as tandem repeats in cell surface proteins and in the extracellular regions of some cell surface receptors [34,35]. In ticks, Ixofin3D, an *I. scapularis* midgut protein containing a signal peptide, four putative fibronectin III domains and a transmembrane protein were shown to play a role in the aggregation of *Borrelia burgdorferi* on the gut epithelium [36]. Silencing of other fibronectin type III domain-containing proteins expressed in the gut of *Anopheles arabiensis* mosquitoes disrupted gut homeostasis following feeding and reduced mosquito longevity [37]. Taken together, it is tempting to speculate that MG6 could also act as a modulator for the bacterial population structure in the tick gut, whereby silencing of MG6 could lead to reduced feeding success and increased tick mortality due to disruption of the gut homeostasis. Additional studies will be required to examine the physiological function of MG6 and its potential as a tick-protective antigen within an anti-tick vaccine in more detail.

Although the injection of dsRNA in the haemocoel of ticks usually results in a systemic RNAi response, the RNAi efficiency may vary between target genes and experiments. In our study, silencing levels ranged from ~96% in the expression of MG9 (A0A131YAQ2) to a complete absence of gene silencing in ticks injected with MG11 (A0A131XS30)-dsRNA, suggesting that MG11 is a refractory target gene for RNAi (Figure 5). In insects, other factors than the target gene, such as the targeted species, strain, tissue and life stage have also been reported to play a role in RNAi efficiency [38]. These and other factors, such as the optimal dsRNA amount for RNAi, have not yet been systematically investigated in ticks, although differences in RNAi efficiency between tick cell lines have been reported [39].

Significantly lower engorgement weights were found for the MG4-silenced females, despite a limited reduction (~31%) in MG4 transcript levels. MG4 is a putative ADP/ATP translocase also known as the adenine nucleotide translocator (ANT) protein, which exchanges ADP/ATP through the mitochondrial inner membrane and is essential for the cellular energy metabolism. In most eukaryotes, multiple ANT proteins are present, with some paralogs being exclusively expressed in testicular germ cells, where they are thought to be essential for spermatogenesis by supplying meiotic cells with ATP [40]. The ubiquitous expression of MG4 and its RNAi phenotype suggest that this protein has a critical role in organismal homeostasis. The high level of amino acid sequence homology of vertebrate and arthropod ANTs [40] may however limit the usability of this protein as an anti-tick vaccine antigen.

Significantly lower engorgement weights were also found for MG9-silenced females. Since the coding sequence for MG9 (A0A131YAQ2) does not contain a stop codon, it is likely to be a truncated version of *I. ricinus* protein V5ICT5, with which it shares 99% AA sequence identity. V5ICT5 is an uncharacterized protein of 1003 amino acids with a predicted mass of 113 kDa. It has a signal peptide, three apple domains, and a transmembrane domain. MG8 (A0A147BMG4), the silencing of which led to a significant reduction in females that fed successively but had no effect on the engorgement weight, has a similar structure with a signal peptide, four apple domains as well as a transmembrane protein. Apple domains are characterized by six cystine residues at highly conserved positions that through the formation of disulfide bonds form a structure which resembles an apple when drawn [41]. These domains are also present on plasma proteins such as factor XI and prekallikrein, where they are essential for binding of substrates [42,43]. The exclusive expression of MG8 and MG9 in the tick midgut, their upregulation upon feeding, and RNAi phenotypes warrant further studies into the function of these proteins.

The proportion of ticks that were successfully engorged was reduced in the MG2 (V5IFB6) and MG10 (V5H492)-silenced females, homologs of the integrin beta and integrin alpha subunits, respectively. Integrins function as cell surface receptors, providing a transmembrane link between the extracellular matrix and the cytoskeleton. Silencing of the expression of integrin beta subunits also had detrimental effects on the development of other arthropods such as the Oriental tobacco budworm, *Helicoverpa assulta*, and the beet armyworm, *Spodoptera exigua* [44,45].

## 5. Conclusions

Co-immunoprecipitation of tick tissue extracts with bovine immune serum raised against these extracts followed by LC-MS/MS analysis led to the identification of immunodominant proteins. This included several proteins that had previously raised interest as potential anti-tick vaccine antigens. Gene silencing of seven out of 11 selected immunodominant proteins targets resulted in a significantly decreased engorgement weight (MG4, MG6, MG9, and SG2) and/or a significant reduction in the number of ticks that successfully engorged (MG2, MG6, MG8, MG10, and SG2) compared to GFP-injected control groups. Although definite proof in the form of vaccination trials against these proteins remains outstanding, we tentatively conclude that the followed approach may be useful in anti-tick vaccine antigen discovery pipelines.

## Figures and Tables

**Table 1:** Details of the quantitative RT-PCRs performed in this study.

Target	UNIPROT ID	Forward Primer (5′-3′)	Reverse Primer (5′-3′)	Amplicon Length (bp)
ELF1A		CAAGATTGGTGGTATCGGCA	GACCTCAGTGGTGATGTTGGC	106
ATP5L		CGAAAGCCATCACTACCCTG	TCTCCACCTTGGCATACTTGAC	85
MG1	A0A131XWI1	GGATGTGGTTTTACCCCGTC	CCCTCTTGAGCGTTGGATG	115
MG2	V5IFB6	CTCTGGAAACCTGGACAACG	GCAGCGTGAAAGATAGAGTCC	131
MG3	V5HWP4	AATCGCCAGTTGTCAGAAGC	TCAAGCCGACAGCAAATATG	76
MG4	V5I2L3	CACTGGTCATCTCCTGGCTC	CGTGCTCTTGTACATAAGGTCTG	137
MG5	A0A0K8R8I3	GTCTCTGCTTCGGTGTCTCC	GGCGACTTGAGGTTGTAGG	83
MG6	A0A147BXB7	GAGACTCCCAAGGACAAGAACC	TGTAGAGATATTTTTGCCACCAGG	78
MG7	V5IJN2	CCGAAGTCTCCAAGGGTCC	ACCGACTCCATCGTCAAAAAG	116
MG8	A0A147BMG4	GACAACACCACGGCACAGG	GGTGTAGGGCTTGAAGTTGTAGAA	92
MG9	A0A131YAQ2	GGGGATTTCCGAAGCCAC	CTGAAGATATTGTTGACGGGGTC	146
MG10	V5H492	AAACGGGCATCAGCAAAGC	TTGTTGAGATCGCCAGCAGAC	97
MG11	A0A131XS30	CATTCGTAGATCACACCCTGC	CGGCGATTCGTAGCGTG	107
SG1	V5HWD5	CCACTACGAAGGCTACCACAA	CCTATTCAGCCCTGTCCATC	56
SG2	A0A0K8RKT7	TTGCCTACGAGATGCTGTCC	TGAACTTGTCCGACTTGAGGT	135
SG3	A0A0K8RPW5	AGTTTACGAGCTTCTCTTGCC	TCCGTCGTGAACACTACCG	102
SG4	A0A0K8RQF1	CTTCCGAAGAGTGTCAGGGTGA	GTGCCGAATGCCGACTGC	108

**Table 2:** List of primers used for dsRNA synthesis. All primers contained a T7 promoter sequence (5′-TAATACGACTCACTATAGG-3′) on their 5′ end. GFP: green fluorescent protein.

Target	Forward Primer (5′-3′)	Reverse Primer (5′-3′)	Amplicon Length (bp)
MG1	T7-CCCTTCCATCTTGCGGTAGC	T7- CGAACGAAGAGCGGAACG	393
MG2	T7-GAATCCCCAGTCCAAGATGATC	T7- CTTTCGTGACCGCTCGTTC	538
MG4	T7-CAGACATCGGCAAGGGTG	T7-GAGCCAGGAGATGACCAGTG	229
MG6	T7-GCGGACGAAGAGGAATACG	T7-GCTAAGAGTAACATTGGTGTATCC	493
MG7	T7-ACCACATCTGCCAACGGAG	T7-ATCCCAAGTAGGAAGCCGTT	257
MG8	T7-ACTTTGCTTTCTTGGCATCGG	T7-GTCGTATGTGTTGCCTTTGTCG	429
MG9	T7-GGTGGCATTGACAACGCTCTC	T7-GAACTTCTTCGTCGCTTCCTTG	400
MG10	T7-GGCTCCAGAAAACACAATCCTC	T7-CCTTTTCCGTGGTAGAATGGG	666
MG11	T7- CCAGGATGGGAAAGTGCGAC	T7- GAACGCCAGCGAACCAGG	243
SG2	T7-CCAAACCTGCCCTACTACCTG	T7-GGACAGCATCTCGTAGGCAAT	324
GFP	T7-GGCCACAAGTTCAGCGTGTC	T7-GCTTGATGCCGTTCTTCTGC	415

**Table 3:** List of proteins identified by LC-MS/MS from *Ixodes ricinus* salivary gland extracts (SGE) following co-immunoprecipitation with antisera raised against SGE. The Mascot score reflects the combined scores of all observed mass spectra that matched to amino acid sequences within the respective protein. MW: molecular weight.

Uniprot Accession Code	Protein Name	# of Unique Peptides	Mascot Score d0	Mascot Score d68	MW (kDa)	pI	Signal Peptide
V5H4T2	Trifunctional purine biosynthetic protein adenosine-3 *	1	41.20	71.91	7.3	8.19	No
V5HMC9	Small nuclear ribonucleoprotein G, putative	1		54.37	8.4	8.54	No
V5IF42	Myosin-2 essential light chain	1	40.41		9.7	6.51	No
V5HG94	60S ribosomal protein L22	2	76.25	88.50	9.9	10.01	No
A0A0K8RQM9	Small nuclear ribonucleoprotein Sm D3	1		58.85	13.2	10.13	No
V5IJC3	60s ribosomal protein L11	1	128.84	79.57	13.2	10.62	No
A0A0K8RIJ1	Histone H2A	1		50.33	13.4	10.73	No
A0A131Y512	40S ribosomal protein S16	1		32.98	14.9	10.04	No
V5HG43	Stromal cell-derived factor 2, putative	1	34.15		15.2	10.32	No
A0A090XEK9	Myosin, essential light chain	2	41.67	44.63	15.5	4.94	No
V5HWD5	Metalloproteinase **(=SG1)** ^a^	1		44.26	16.9	9.92	No
A0A0K8RC23	40S ribosomal protein S13	1		33.57	17.2	10.68	No
A0A0K8RL33	Superoxide-dismutase	1		35.85	18.1	6.64	Yes
V5HD78	60S ribosomal protein L6	4	62.85	57.09	18.6	10.33	No
V5I150	60S ribosomal protein L5-A	3	94.73	50.00	19.0	7.84	No
V5I135	Alpha-crystallin A chain *	1	54.14		20.3	7.64	No
V5HXA8	60S ribosomal protein L18	2	60.03	131.89	21.5	11.62	No
V5H3S3	60S ribosomal L23	2		67.50	21.6	11.39	No
A0A0K8RQ35	40S ribosomal protein S8	1		40.95	21.9	10.30	No
A0A0K8RKT7	Glutathione S-transferase **(=SG2)**	1		39.95	25.5	7.88	No
A0A0K8RPW5	Metalloproteinase **(=SG3)** ^a^	1		45.77	26.6	9.45	Yes
V5I164	Tropomyosin, isoform close to X4	3	145.00	57.96	26.6	5.34	No
A0A0K8RHG9	Tubulin alpha chain	1		62.27	27.1	5.57	No
A0A0K8RQF1	Toll-like receptor, putative **(=SG4)**	1		41.27	27.6	8.31	Yes
A0A131XW65	60S ribosomal protein L7	1	79.15	70.36	29.2	10.98	No
A0A0K8RG40	40S ribosomal protein S4	3	74.40	81.92	29.6	10.29	No
A0A0K8RG01	Glyceraldehyde-3-phosphate dehydrogenase 2, isoform X1 *	1		51.70	36.0	7.84	No
V5HG89	ATP synthase subunit beta	2		87.40	36.3	5.03	No
E3SS18	Translation elongation factor EF1-alpha *	4	63.01	184.99	36.7	8.27	No
A0A147BVX5	Venom metalloproteinase antarease-like TtrivMP_A	1	34.45		38.8	5.83	Yes
Q5D579	Actin *	9	401.51	335.42	41.5	5.85	No
A0A0K8RDN7	Protein N-myc downstream-regulated gene 3 (NDRG3) isoform X1	2	46.54	88.18	44.6	6.54	No
A0A0K8RCY6	Tubulin beta chain	3	40.59	127.53	45.1	5.97	No
A0A131XPA0	Eukaryotic translation initiation factor 3 subunit M	1		36.48	45.2	5.97	No
A0A0K8RMJ6	60S ribosomal protein L4	1		37.81	46.6	11.19	No
V5I085	Microsomal triglyceride transfer protein large subunit ^b^	1	36.01	58.33	48.0	6.89	No
A0A0K8R4C2	Dolichyl-diphosphooligosaccharide–protein glycosyltransferase 48 kDa subunit	1		37.19	48.8	6.05	Yes
A0A0K8R4D7	Cytochrome b-c1 complex subunit 2	3	105.77	115.65	48.9	8.62	No
A0A131Y1S2	Microsomal triglyceride transfer protein large subunit ^b^	2	36.65	51.53	49.7	8.97	Yes
V5I095	S-adenosylhomocysteine hydrolase-like protein	4		93.01	50.5	6.54	No
A0A131XNF3	Processing peptidase beta subunit, putative *	2	105.08	87.93	53.4	6.15	No
A0A0K8RCY2	Metis1	4	149.35	176.93	55.5	7.58	Yes
A0A131XPM3	Heat shock protein 60 *	5		151.93	59.3	5.62	No
A0A0K8RCE8	Heat shock 70 kDa protein cognate 4 *	2	60.97	115.44	59.8	7.43	No
A0A0K8RP16	Dolichyl-diphosphooligosaccharide--protein glycosyltransferase subunit 1	3		115.81	67.5	7.53	Yes
A0A090XC63	Moesin/ezrin/radixin homolog 1 isoform X1 *	7	81.17	206.20	70.1	5.66	No
A0A0K8RIU3	Heat shock protein, putative *	3	66.94	102.00	72.6	5.41	Yes
V5HP83	Coatomer subunit alpha	2		61.57	75.6	8.79	No
A0A0K8R8N9	Heat shock protein HSP 90-alpha	1		37.81	84.2	5.02	No
V5HRY6	Sodium/potassium-transporting ATPase subunit alpha-B	3		144.31	91.7	5.16	No
A0A131XXE4	F-box only protein 11	1	37.74		99.1	7.06	No
A0A131XWG4	Coatomer subunit beta	2		59.80	103.2	5.21	No
V5GY25	Clathrin heavy chain 1 *	3	35.11	119.33	190.7	5.81	No
V5I4B8	Myosin heavy chain, muscle isoform X3 *	40	1378.54	1750.99	222.0	6.09	No
V5I3C9	Myosin heavy chain, non-muscle isoform X1 *	15	354.64	650.15	227.5	5.55	No

* indicates a protein identified by LC-MS/MS in both ME and SGE following co-immunoprecipitation with antisera raised against ME and SGE, respectively. Entries with the same superscript letter in the protein description ^a,b^ represent different fragments of the same protein.

**Table 4:** List of proteins identified by LC-MS/MS from *Ixodes ricinus* midgut extracts (ME) following co-immunoprecipitation with antisera raised against ME. The Mascot score reflects the combined scores of all observed mass spectra that matched to amino acid sequences within the respective protein. MW: molecular weight.

Uniprot Accession Code	Protein Name	# of Unique Peptides	Mascot Score d0	Mascot Score d68	MW (kDa)	pI	Signal Peptide
V5H4T2	Trifunctional purine biosynthetic protein adenosine-3 *	1	52.26		7.3	8.19	No
V5HY31	Histone H4-like, putative	1		37.81	9.8	10.43	No
A0A0K8RK48	Uncharacterized protein	1	43.68	44.01	13.2	7.83	Yes
A0A0K8RQA6	Ubiquitin	1	47.08		14.5	9.82	No
V5H0K4	Pantetheinase, putative ^a^	1		161.39	16.3	6.54	Yes
A0A131Y7G4	Uncharacterized protein ^b^	1	58.35	84.84	18.2	8.29	Yes
V5I2L3	ADP/ATP translocase, putative **(=MG4)**	1		42.62	19.3	9.58	No
V5I135	Alpha-crystallin A chain *	1	110.22		20.3	7.64	No
A0A090X8W5	Uncharacterized protein ^b^	1	73.12	61.59	21.7	8.94	Yes
A0A131YAP7	Tropomyosin isoform X15/X16	1	61.58		23.6	4.74	No
V5HHC0	Uncharacterized protein	1	58.35	95.16	23.8	9.85	Yes
A0A131XX88	60S ribosomal protein L19	1	44.69		24.2	11.43	No
A0A131XRL8	Cathepsin L	1		72.31	27.7	5.48	No
A0A131XWI1	Salivary secreted cytotoxin, putative **(=MG1)**	1		66.40	32.0	9.33	No
V5HBQ2	Lysosomal Pro-X carboxypeptidase	1		40.01	33.9	5.08	No
V5HWP4	Uncharacterized protein **(=MG3)**	1		48.01	35.0	7.01	No
A0A0K8RNA0	Malate dehydrogenase	2	73.48		35.8	9.09	No
A0A0K8RG01	Glyceraldehyde-3-phosphate dehydrogenase 2 isoform X1 *	1	39.91		36.0	7.84	No
E3SS18	Translation elongation factor EF1-alpha *	3	54.98	36.81	36.7	8.27	No
Q5D579	Actin *	7	248.34	38.91	41.5	5.85	No
A0A0K8RCB1	Enolase	1	38.63		47.1	6.01	No
A0A131XPI3	Aminopeptidase, putative W07G4.4	1		34.48	47.6	8.32	No
A0A147BSS4	Pantetheinase ^a^	1		206.82	52.5	7.01	Yes
A0A131XNF3	Processing peptidase beta subunit, putative *	1	50.89		53.4	6.15	No
V5HEY6	Alpha-L-fucosidase	1		55.17	53.5	6.92	Yes
V5HB74	Retinal dehydrogenase 1	4	181.55	90.92	54.6	6.89	No
V5IJN2	Calcium-activated chloride channel regulator **(=MG7)**	1		39.96	55.1	4.96	No
A0A131Y0J3	Alpha-aminoadipic semialdehyde dehydrogenase	1	39.78		58.7	6.79	No
A0A131XPM3	Heat shock protein 60 *	1		89.26	59.3	5.62	No
A0A0K8RCE8	Heat shock 70 kDa protein cognate 4 *	3	171.54		59.8	7.43	No
A0A131XQI6	Moesin/ezrin/radixin homolog 1 *	1	97.17		62.5	5.52	No
V5HN24	Beta-hexosaminidase subunit beta	1		38.14	63.4	5.40	No
A0A0K8RIU3	Heat shock protein, putative *	3	179.61		72.6	5.41	Yes
A0A0K8R8I3	Uncharacterized protein (cubilin-like?) **(=MG5)**	2		80.81	75.8	6.67	Yes
V5IFB6	Integrin beta-PS (**=MG2**)	3		193.69	83.8	5.17	No
V5GPX7	Alpha-actinin isoform X2	1		38.52	89.4	6.13	No
A0A131YAQ2	Uncharacterized protein **(=MG9)**	1		71.18	94.3	6.62	Yes
A0A147BXB7	Cell adhesion molecule, putative **(=MG6)**	3		70.59	104.8	6.34	Yes
A0A147BMG4	Uncharacterized protein **(=MG8)**	1		34.22	109.1	5.95	Yes
A0A0K8RQE7	Lysosomal alpha-mannosidase-like	1		62.70	109.3	7.56	No
V5H492	Integrin alpha-PS1 **(=MG10)**	1		43.73	110.3	6.14	No
V5H7Z4	Alpha-2-macroglobulin-like protein	2		114.53	152.0	5.59	Yes
V5GY25	Clathrin heavy chain 1 *	1	47.55		190.7	5.81	No
V5I4B8	Myosin heavy chain, muscle isoform X3 *	6	178.22	52.04	222.0	6.09	No
V5I3C9	Myosin heavy chain, non-muscle isoform X1 *	6	68.25	71.36	227.5	5.55	No
A0A131XS30	MAM and LDL-receptor class A domain-containing protein 1 **(=MG11)**	1		40.27	420.4	5.49	No

* indicates a protein identified by LC-MS/MS in both ME and SGE following co-immunoprecipitation with antisera raised against ME and SGE, respectively. Entries with the same superscript letter in the protein descriptions ^a,b^ represent different fragments of the same protein.

## Data Availability

All the data are included within the article.
